# Salicylic Acid

**Published:** 1877-05

**Authors:** 


SALICYLIC ACID.—Mr. J. A. Erskine Stuart has published (*Edinburgh Med. Journal*, November, 1876) some experiments which he has made with this article, and gives the following as his conclusions: "That it is an antiseptic, deodorizer, and astringent, possessing these three properties in a marked manner. That it produces a specific action on the mucous membrane of the mouth, nose, and throat, is undoubted, as the catarrhal symptoms are produced in these regions whether it is taken by the mouth or rectum. That, from its rapid absorption by the blood, it is quickly carried through all parts of the economy, and its action is thus quickly manifested. The antiseptic properties being so marked, and its character being non-poisonous, it is sure to prove efficient in zymotic disease, in the same way that sulpho-carbolate of soda has been used.

"Salicylic acid, although proved by Godeffroy to be three times as strong an antiseptic as carbolic acid, is so difficult to dissolve, that its spray is not efficacious. Its use as an external antiseptic is thus much prevented, as, from its non-irritating, and non-poisonous qualities, it is otherwise eminently suited for use externally. As it is not likely to rise into much fame as an external antiseptic, I shall conclude with a few remarks as to how its action may be explained in three diseases in which it has been used with success, viz., rheumatic fever, typhoid fever, and diphtheria.

"No explanation of the changes which salicylic acid undergoes in the economy has yet been attempted, so far as I have observed in the journals. Is it not probable, as I mentioned previously, that salicylic acid, from the heat to which it is exposed in the alimentary canal, splits into carbolic and carbonic acids; that the carbonic acid, escaping as it passes down, causes the sensation of choking; and that the carbolic acid, coming in contact with gastric juice, forms an innocuous compound with some of its salts, and thus carries out its actions? This is the chemical explanation of its action, at least. By using the prescription before mentioned, combining the potash and salicylic acid, we get rid of the carbonic acid gas, and thus prevent much of the burning in the throat.

"*Rheumatic Fever.*—No doubt is expressed among practitioners as to the beneficial action of this drug in rheumatic fever It has been tested largely in both hospitals and private practice, and found not only to shorten the disease, but also to lower temperature and relieve pain. Cases treated with continuous small doses are soon better. Its action in this disease is antipyretic. Possessing many of the properties of quinine, in the form of salicine, the temperature is lowered much in the same way as a large dose of quinine would do.

"*Typhoid Fever.*—In this disease, salicylic acid has been little used in this country, but from German sources we have reliable information regarding its application. Dr. Reiss, of Berlin, used as large a dose as four scruples. He found that often after the first dose, and usually after the second, the temperature fell below the normal, and remained so for twenty-four hours. In these cases, only one daily dose of the under-mentioned formula was given, and it was found that eight or ten doses were sufficient. The formula for a dose was as follows:—

"℞. Acid. salicyl.; Sodæ carb. aa ℈ iv; Tinct. aurant. ʒj; Aquæ ℥iss. M.

"The result was that, generally after the fourteenth day of the fever, there was no abnormal rise of temperature. Dr. Reiss found that this result happened in the most of 260 cases which he treated.

"The action in typhoid fever may be explained thus: It acts in such enormous doses as an excellent astringent on the bowels, and also as an antiseptic on the system generally, as well as on the bowels.

"*Diphtheria.*—Wagner used it as a remedial agent in diphtheria, and also in an epidemic of it, with success. Frontheim also used it as a prophylactic in this disease, and it seems to me that it will be yet much used as a prophylactic in all febrile disease. My theory of its action in diphtheria is this, that it acts on the body generally as an antipyretic, and that, by setting up its specific catarrhal action on the mucous membranes of the throat, it helps to arrest the disease.

"In the *Edinburgh Medical Journal*, 1837, Dr. Blom ascribes the beneficial action of salicine to the fact that it acts as a tonic to the mucous membranes, and improves the character of the secretions. If this is the case, it may account in some manner for the action of salicylic acid in typhoid fever and diphtheria. Salicine is not so burning a substance to swallow as the salicylic acid, and it does not set up the characteristic catarrh which I mentioned."—*Am. Jour. Med. Sciences.*

